# Molecular Analysis and Ex Vivo Infectivity of Seronegative Occult Hepatitis C Virus: A Study in Single Haemodialysis Centre

**DOI:** 10.21315/mjms2024.31.2.4

**Published:** 2024-04-23

**Authors:** Siti Nurul Fazlin Abdul Rahman, Hairul Aini Hamzah, Mohammed Imad A. Mustafa Mahmud, Noraihan Mat Harun

**Affiliations:** 1 Microbiology Unit, Department of Basic Medical Sciences, Kulliyyah of Medicine, International Islamic University Malaysia, Pahang, Malaysia; 2 Molecular and Biochemistry Unit, Department of Basic Medical Sciences, Kulliyyah of Medicine, International Islamic University Malaysia, Pahang, Malaysia

**Keywords:** hepatitis C virus, patients, PBMCs, sequence analysis, genotype

## Abstract

**Background:**

In occult hepatitis C virus infection (OCI), hepatitis C virus ribonucleic acid (HCV RNA) is detectable in peripheral blood mononuclear cells (PBMCs) but is not evident in serum or plasma. Understanding of OCI in patients with seronegative anti-HCV antibodies is limited.

**Methods:**

In this study, six HCV isolates from haemodialysis (HD) patients with seronegative OCI were identified by molecular assays and phylogenetic analysis. The virus infectivity was assessed ex vivo using a primary naïve PBMC culture system. HCV isolates obtained from the PBMCs of 10 patients with chronic HCV infection (CCI) were characterised concurrently and used as positive controls in the cell culture.

**Results:**

Sequence analysis of the 5′ untranslated region (UTR) and non-structural 5B (NS5B) region revealed that HCV genotype 3 was the most prevalent virus type in both the OCI and CCI groups. One of the occult HCV isolates was identified as a mixed type. The mean viral load (log_10_ RNA copies/106 cells) in the PBMC samples of the OCI group (M = 3.4, SD = 0.7) was lower than that of the CCI group (M = 4.6, SD = 1.7). Upon culture, de novo OCI-HCV replicates were detected in five out of six naïve PBMC cultures. Analysis of the replicates showed a single guanine addition in the domain III of 5’-UTR but the overall molecular structure was retained.

**Conclusion:**

Seronegative OCI is an active form of infection that replicates at a low level in PBMCs. Seronegative OCI may share the same route of transmission as CCI. The retained viral competency may have an implication for its persistence.

## Introduction

Hepatitis C virus (HCV) is an agent of chronic liver disease that may lead to cirrhosis and liver carcinoma. The virus has a single-stranded RNA genome of 9.6 kilo base pair, which is flanked by the untranslated region (UTR) at both 5’ and 3’ ends. The 5’UTR is commonly targeted by molecular assays as it shares more than 90% of sequence identity across different HCV genotypes. Due to a lack of proofreading properties of the virus RNA polymerase, mutation leads to diverse HCV genotypes and subtypes. Recently, eight HCV genotypes have been identified (1). Each genotype is further classified into subtypes with a sequence divergence of > 15% (2). Besides being a key tool in controlling therapeutic regimens, HCV genotyping provides insight into virus transmission; hence it is essential in the control programme (3).

In haemodialysis (HD) settings, the prevalence of HCV infection among patients remains higher than in the general population. This is due to the requirement for blood transfusions, which is one of the main routes of HCV transmission. Furthermore, patients on regular HD commonly develop uraemia, a condition which impairs their immune functions and increases susceptibility to infection (4). Recently, an increasing number of studies on a silent form of HCV infection known as occult HCV infection (OCI) have been reported (5–9). OCI is characterised by the presence of HCV RNA in peripheral blood mononuclear cells (PBMCs) and the absence of HCV RNA in serum or plasma regardless of their serostatus of anti-HCV antibodies. OCI with negative anti-HCV antibodies is called ‘seronegative OCI’. The ability of HCV to establish silent infection in PBMCs raises the question of what the characteristic features of occult HCV are.

Not only detectable in PBMCs, accumulated evidence indicates that HCV also replicates in the subsets of lymphocytes (10, 11). The studies demonstrated HCV replication through the detection of RNA replicative intermediate known as the ‘negative-strand’ or ‘anti-genomic strand’ in PBMCs. The anti-genomic strand serves as the replicative template for producing more genomic positive strands, which encodes a single polypeptide that is subsequently cleaved and post-translationally modified to at least 10 structural and non-structural proteins (12).

Patients with chronic HCV infection (CCI) commonly manifested with HCV RNA in PBMCs, however, it is not clear if HCV found in PBMCs of patients with seronegative OCI is competent enough to cause persistent propagation in the cells. Furthermore, the clinical significance of OCI has been uncertain since its first description (12–14). In our preliminary study, we found the existence of seronegative OCI cases in an HD referral centre. Therefore, the main objectives of the present study were to determine the molecular characteristics of occult HCV and its replication status in PBMCs as well as to assess the virus infectivity in an ex vivo system.

## Methods

### Patients

This cross-sectional study was conducted at the main HD centre in Kuantan City, Pahang, Malaysia. This centre was one of the referral centres for dialysis management in East Coast Malaysia. HCV screening was conducted once every 3 months in accordance with the standard operating procedure in HD provided by the Ministry of Health, Malaysia. Patients with chronic HCV infection (CCI) were compartmentalised from those who were clinically free from HCV. All of them were undergoing direct-acting antiviral-based regimens at the time of the present study.

The minimum sample size was calculated using the following formula:


$$n=\frac{Z^{2}P(1-P)}{d^{2}}$$

where *n* = sample size; *P* = expected prevalence; *Z* = statistic corresponding to the level of confidence; *d* = precision.

The prevalence of OCI in Malaysia is unknown. In Thailand, the proportion of OCI is thought to be around 18% (8). With the desired precision of 0.2 and a confidence level of 95%, the minimum sample size required is 15 dialysis patients.

The inclusion criteria for patients eligible for the study were that they were more than 18 years old and were not infected with either the human immunodeficiency virus or the hepatitis B virus. Patients provided written informed consent for sample collection and for the anonymous use of data for research purposes. Upon consent, patients’ demographic and baseline clinical characteristic data were obtained from their medical record files. Ten patients with known CCI and 30 clinically non-infected HD patients, who were persistently negative for antibodies in their serum, were enrolled in the study. Blood samples from 10 healthy non-HD individuals were also collected for negative controls in the molecular assays and cell cultures. Before the study began, the study protocol was approved by the Medical Research Ethics Committee (MREC), Ministry of Health Malaysia and the International Islamic University Malaysia (IIUM) Research Ethics Committee.

### PBMCs and Virus Isolation

PBMC samples were isolated from 10 mL of blood using the Ficoll-Hypaque density gradient technique (GE Healthcare, USA). Cells were washed three times with Mg^2+^ and Ca^2+^ free phosphate buffer saline (PBS) before being resuspended to 1 × 10^6^ cells/mL in Roswell Park Memorial Institute (RPMI) 1,640 tissue culture medium (Thermo Fisher Scientific, USA) containing 10% foetal bovine serum (FBS) (Sigma-Aldrich, USA) and antibiotics (penicillin/streptomycin, 10 mg/mL). Isolated cells were subjected to cell counting and a viability assay using the trypan blue exclusion method.

### Reverse Transcription and Polymerase Chain Reaction Amplification

Total RNA from PBMCs was extracted using SV Total RNA Isolation System (Promega, Madison, WI, USA). The synthesis of cDNA was accomplished using a reverse transcription system (Promega, Madison, WI, USA) with a random primer. Polymerase chain reaction (PCR) was performed using established primers that hybridised to the highly conserved 5’UTR of the viral genome to detect the genomic (15) and anti-genomic (14) strands of the virus (Table 1). Samples with unsuccessful cDNA amplification in the first round of PCR were subjected to a semi-nested (15) or nested (14) PCR detection procedure, depending on the virus genomic strands targeted. Primers targeting the non-structural 5B (NS5B) region were used for subtyping purposes (16).

Amplification of the genomic cDNA was performed using GoTaq^®^ DNA polymerase (Promega, USA) in a total volume of 25 μL. The thermal profiles for the amplification of both strands were as follows: initial denaturation at 95 °C for 1 min; 40 cycles with denaturation at 95 °C for 30 s, annealing at 56 °C for 30 s and elongation at 72 °C for 30 s. The final extension was set up at 72 °C for 5 min. Semi-nested and nested PCRs were performed in the same way as in the first round except for different reverse primers. The same procedure was done for anti-genomic amplification (14).

### DNA Sequencing and Typing

All PCR products were purified using a MinElute gel extraction kit (Qiagen, Valencia, CA, USA). The purified PCR products were then sequenced bidirectionally using the Sanger sequencing method and the same PCR primers. Sequences were compiled and a nucleotide similarity search was conducted using the Basic Local Alignment Search Tool (BLAST, https://blast.ncbi.nlm.nih.gov/Blast.cgi) to confirm the identity of the gene segment. Thereafter, nucleotide sequences were aligned by ClustalW alignment using the MEGA 4.1 software package (www.megasoftware.net) (17) together with known sequences obtained from the National Centre for Biotechnology Information (NCBI) homepage (https://www.ncbi.nlm.nih.gov/nuccore). For HCV typing, phylogenetic trees were constructed to distinguish each HCV sample into different genotype and subtype groups. Pairwise evolutionary distance matrices for the nucleotide sequences were computed using the JukesCantor algorithm and the neighbour-joining method for tree drawing.

### Real-Time PCR Quantification Assay

We used a GoTaq^®^ qPCR (Promega, USA) kit to quantify the HCV RNA in the cultured cells. The synthetic DNA fragment of 5′UTR HCV (gBlock, IDT Integrated DNA Technologies, Singapore) with a known concentration was used as a standard by preparing a serial 10-fold dilution. The real-time PCR assay was run on a CFX-96 real-time system (C1000 Touch, Bio-Rad, USA). The primers for genomic strands were used in the quantification assay with a similar amplification profile to that of as the conventional PCR. The melting point was determined by a gradual decrease in the temperature, dropping from 95 °C to 65 °C. The detection limit of the assay was 1 log_10_ RNA copies (80 RNA copies/mL).

### Preparation of Inocula for Cell Culture

PBMCs were cultured under the optimised conditions in T75 cell culture flasks. They were incubated within 2 days to 4 days in a 37 °C humidified incubator in an atmosphere of 5%–7% CO2. The cells in suspension were centrifuged at 150 × g for 10 min at 4 °C. Then, the supernatant was discarded, and 15 mL of fresh culture media was added into the tube and prepared for subculture in new T75 flasks. The PBMCs were then incubated in a similar condition as mentioned before. Phytohaemagglutinin (PHA) was added to the culture medium at a concentration of 5 μg/mL and incubated over a period of 48 h–72 h to stimulate cell growth. Thereafter, the supernatant of each PBMC sample was collected as inoculum in the infectivity testing.

### Preparation of Naïve Peripheral Blood Mononuclear Cells and Infectivity Testing

HCV infectivity was determined using previously established methods (6, 18). PBMC samples collected from the healthy non-HD individuals (negative control group) were pooled into a tube. The cells were then treated with 5 μg/mL of PHA for 48 h prior to the inoculation procedure. After 48 h of incubation, the cells were washed three times with PBS and cultured in RPMI medium containing 20% FBS and 10% recombinant IL-2 for 72 h until it reached 1 × 107 cells/mL. Then, the cells were prepared for seeding by being aliquoted in triplicates into 24-well plates, with each well containing 1 × 106 cells/mL (Appendix Figure 1). Thereafter, the cells were inoculated with 250 μL of the prepared inocula and agitated gently for 4 h, at 37 °C. The inocula were removed after 24 h, and the cells were washed and cultured in a 37 °C CO2 incubator. Media and cells were observed daily. At 14 days post-inoculation, the cells were counted before being harvested and washed three times. RNA was extracted from the cells for molecular testing.

### Secondary Molecular Structure and Statistical Analysis

The obtained DNA base sequences of 5’UTR were aligned using BioEdit software, as mentioned before, along with the reference sequences from the NCBI website database. The secondary two-dimensional structure was predicted by analysing the sequence on the Mfold programme freely available online (http://www.unafold.org/mfold/applications/rna-folding-form.php).

IBM SPSS software for Windows version 28.0 was used for statistical analysis. Continuous variables were expressed as mean (standard deviation [SD]) and categorical data were represented by ratio. One-way ANOVA and Kruskal-Wallis H test were used to compare means between two variables of more than two categories and the Mann-Whitney U test for two categories. The differences in data results were considered statistically significant if the P was < 0.05 (two-tailed test, 95% confidence interval).

## Results

### Detection of Genomic and Anti-Genomic Strand HCV RNA in PBMC

To confirm the presence of HCV in the PBMC samples, primers targeting the most conserved region of the HCV genome were applied using the 5’UTR-based PCR amplification method, which detected genomic viral HCV RNA in 10/10 of the PBMC samples of the CCI group and 6/30 PBMC samples of seronegative patients by the detection of 212 base pair (bp) (first round PCR) or 153 bp (semi-nested PCR) of the targeted HCV-5’UTR. Herein, six of these seronegative samples were grouped as OCI. Similar to the detection methods of genomic HCV strands, the 5’UTR-based assay was used for the detection of anti-genomic strands. Active replicates were detected in all (10/10) PBMC samples of the CCI group and all six PBMC samples of OCI by nested PCR, which resulted in 266 bp bands (Figure 1).

### Patients’ Data Analysis

Patients were categorised into three groups for data analysis based on the HCV infection status (Table 2). Men were dominant in all categories. There was no significant difference in liver enzymes, haemoglobin and creatinine levels, but there were significant differences in the ages (*P* = 0.019) and duration of dialysis (*P* = 0.007). Further analysis of the duration of dialysis using the nonparametric post-hoc test showed a significant difference between the non-infected group and CCI, but not with OCI or between CCI and OCI. Regardless of patients’ serostatus, most patients had high serum creatinine concentrations as expected. Normal alanine transaminase (ALT) levels were observed in all patients with CCI. However, a raised ALT level was documented in two of the patients with OCI and their aspartate transaminase (AST) levels: ALT ratios were less than 1 (Table 3).

### HCV Type and Viral Loads

**Figure 2:**
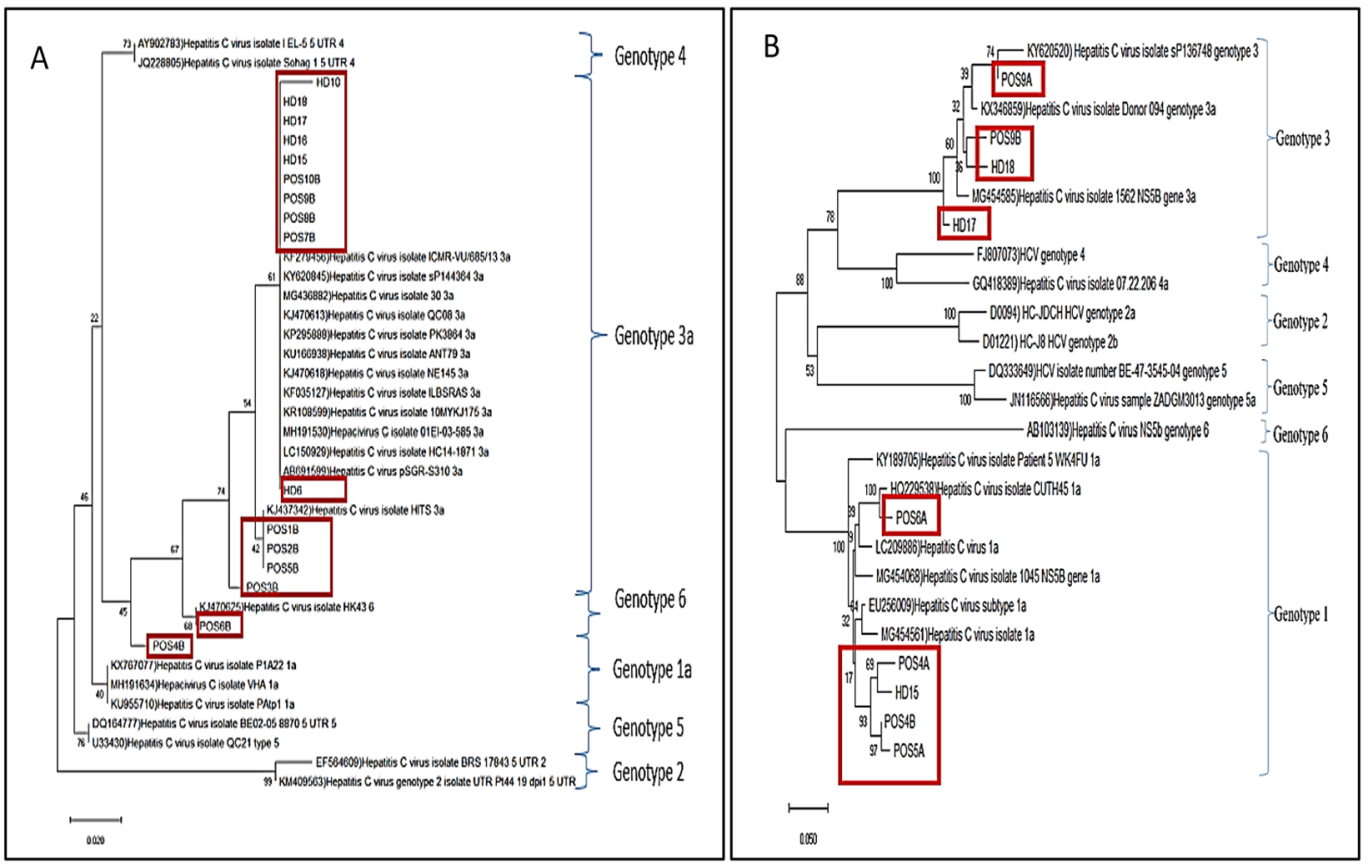
Phylogenetic tree of the nucleotide HCV sequences. (A) Phylogenetic analysis based on the 5′UTR sequences shows HCV genotypes 3, 1 and 6 in patients infected with chronic or occult HCV (boxes). (B) Subtyping the HCV infecting PBMCs based on the NS5B phylogenetic analysis reveals subtypes 3a and 1a (boxes). The isolates’ nomenclature: the prefix ‘POS’ of the virus isolates in this study indicates chronic HCV isolates, meanwhile, the prefix ‘HD’ indicates occult HCV. Note that occult HCV isolates are not clustered together suggesting different strains

Phylogenetic analysis of the genomic 5’UTR sequences revealed that the main viral genotypes of the HCV isolates in the CCI group were genotype 3 followed by genotype 1; meanwhile, only HCV genotype 3 was found in the OCI group (Figure 2A). Genotype 6 was found in 1/10 of the CCI group. For subtyping, the HCV NS5B region from 2/10 of the CCI and 3/6 of the OCI was successfully amplified and analysed, which revealed the subtype 3a and 1a infections in both groups (Figure 2B). NS5B sequences of occult HCV isolates were not in the same phylogenic cluster due to variation of their nucleotide sequences, indicating that the isolates were of different strains (Figure 2B). Based on the two typing approaches, mixed genotypes 3 and 1a were found in one sample of the OCI group (HD15) (Table 3). Viral loads in PBMC samples were determined for both the CCI and OCI groups of patients (Table 3), which ranged from 2.0 to 7.7 log_10_ RNA copies/106 cells. The average level of viral loads in the OCI-PBMC samples was lower (mean = 3.4, SD = 0.7) than that of the CCI-PBMC group (mean = 4.6, SD = 1.7). High viral loads (7.7 log_10_ RNA copies/106 cells) were recorded in one sample of the CCI-PBMC group, which was also the only sample infected with HCV genotype 6.

### Occult HCV Infection in Naïve PBMCs

**Figure 3:**
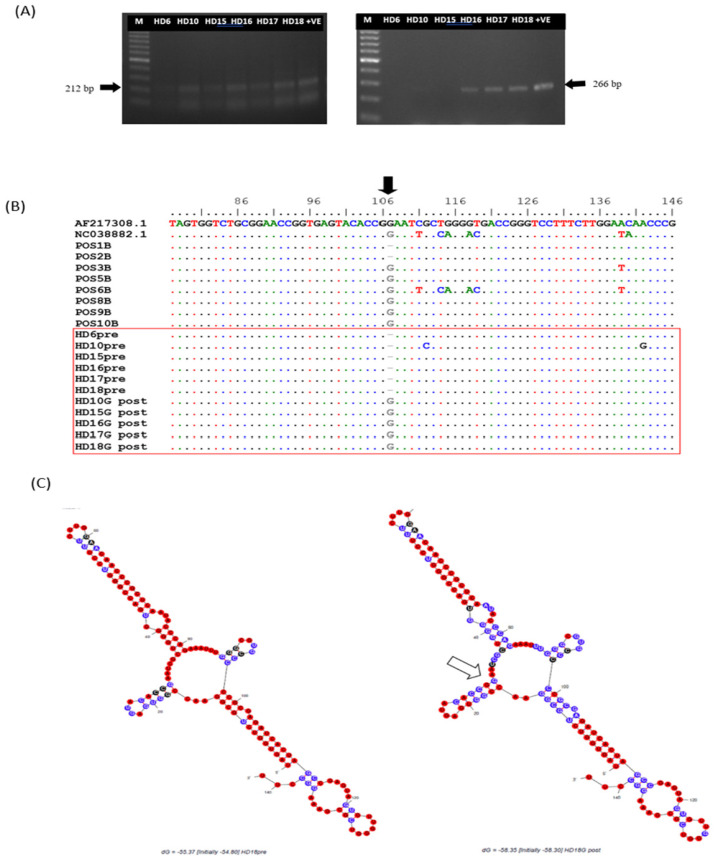
Molecular detection and nucleotide sequence analysis of the de novo occult HCV isolates. (A) Genomic (right) and antigenomic strands (left) of the HCV RNA were detected in the respective naïve PBMC cultures exposed to the supernatants of PBMC cultures infected with six occult HCV isolates. Supernatant derived from a PBMC culture infected with chronic HCV was run in parallel as the positive control. M = 100 bp ladder (New England Biolabs, USA), +VE = positive control. HCV = hepatitis C virus. (B) Sequence alignment of the HCV isolates shows the addition of guanine (G) nucleotide in the de novo occult HCV sequences (indicated by the black arrow). HCV isolates HD6, HD10, HD15, HD16, HD17 and HD18 with the prefix ‘PRE’ indicates before inoculation, and viral isolates of the post-inoculation are given the prefix ‘POS’. AF217308 is a reference sequence obtained from the NCBI database. (C) Prediction of the domain-III 5’UTR-HCV secondary structure using HD18 sequence reveals no change to the overall 2-D structure despite the addition of G nucleotide. Notes: White arrow indicates the position of G addition; A = adenine; T = thymine; C = cytosine; HCV = hepatitis C virus

To determine whether HCV recovered from OCI-PBMCs could infect naïve PBMCs, a pool of PHA-treated naïve PBMCs from non-HD individuals in the negative control group was cultured and exposed to the supernatants of the OCI-PBMC culture after a serial passage. As shown in Figure 3A, genomic strands of the HCV RNA (212 bp) were clearly detected in 5/6 of the seeds exposed to the OCI-PBMC supernatants. The intracellular viral load in the PBMCs was estimated to be between 2 and 4 log_10_ RNA copies/105 cells, which could be detected by the first round of PCR. Meanwhile, anti-genomic strands (266 bp), which are the indicators of active replication, could be detected by nested PCR in 3/6 of the seeds (Figure 3A).

### Molecular Analysis of de novo Viral RNAs

To gain insight into the sequence identity of the de novo viral RNAs, the extracted RNA from infected cells was amplified by PCR with the genomic and anti-genomic strand-specific primers. Thereafter, the amplified products were sequenced via direct DNA sequencing. Analysis revealed that all nucleotide sequences of the genomic and anti-genomic strands were clustered along with the genotype 3a reference sequence. Multiple alignments of the sequences were also performed along with reference sequences (Figure 3B). Analysis of the post-inoculated HCV sequences showed an insertion of guanine (G) at position 107 of the domain III 5’UTR compared to the pre-inoculated HCV nucleotide sequences of the same HCV isolates. Using isolate HD16 as the representative, the effect of the G insertion was further analysed by comparing the predicted two-dimensional structures of the pre- and post-inoculated HCV sequences, which revealed no changes in the overall molecular structure (Figure 3C).

## Discussion

The existence of OCI in HD patients continues to be reported especially in regions showing a high prevalence of hepatitis C virus (19). Extensive studies on OCI have been conducted in European countries and the USA (7, 11, 13, 14). A systematic review related to OCI in the Middle East and Mediterranean countries revealed an overall prevalence of 10.04% (19). A prospective study at three dialysis centres in Chiang Mai, Thailand, suggested that OCI may be common among HD patients (8). In Vietnam, a high prevalence of infection involving diverse and novel HCV variants has been reported in dialysis patients (20). Nevertheless, knowledge and epidemiological data on OCI are still lacking among the Southeast Asian population.

We conducted a study on seronegative OCI among HD patients, as there were no available data on OCI among our population. We found that 6 out of 30 seronegative patients (20%) who were clinically free from HCV had viral RNA in their PBMCs. A high prevalence rate in a single centre may indicate an outbreak. However, NS5B region analysis of the OCI HCV isolates suggested that the infections were caused by different strains (Figure 2B). Besides, one of the patients had a mixed-genotype infection (3a and 1a). We have reported in a previous study that HCV genotype 3 was the predominant HCV genotype circulating in our general population, followed by genotype 1 (15). The same genotypes were also found circulating among the HD patients with CCI and OCI, depicting a similar route of HCV transmission in both groups which could be disseminated horizontally across the HD community. Furthermore, the baseline data analysis showed that older patients with longer duration of dialysis were more susceptible to both CCI or OCI, thus associating the two independent variables as risk factors for HCV in the centre. These findings support the possibility that the HD patients had been exposed to HCV through blood transfusions or blood products. The finding concurred with a study involving multi-dialysis centres that confirmed that the administration of blood products is the main risk factor for developing HCV (21). The possibility of blood donors becoming unknowingly infected is unknown. In China, 2.2% of blood donors have OCI (22). However, OCI status in the study patients was not further followed-up and, thus, the persistence of the infection is unknown. In Thailand, a previous study reported findings of HCV RNA detection in PBMCs of 2 out of 11 HD patients who transiently lost detectable HCV viraemia suggesting that resolved OCI may also not be uncommon (8).

Anti-genomic strands serve as the replicative intermediate for HCV replication and therefore confirmed active HCV replication. However, the quantification of anti-genomic strands does not represent the actual viral load in a sample; therefore, genomic strand copy numbers should be used for viral load determination. A viral load of less than 6.3 log_10_ RNA copies/mL was considered low (23) and, based on the cut-off point value, viral loads in the PBMCs of patients with OCI were generally lower than those of patients with CCI. A low viral load usually indicates a better prognosis. The infection either resolves naturally or results in disease reactivation due to impaired cellular immunity (24).

In the current study, PBMCs from patients with OCI and CCI were washed several times and cultured, and the virus released in the supernatants was capable of causing infection in naïve PBMCs from healthy individuals, suggesting that the occult HCV is infective ex vivo. Productive HCV replication was documented by the molecular detection of HCV RNA genomic and anti-genomic strands. The pool of naïve PBMCs used as a substrate for virus replication was freshly isolated from healthy individuals and cultured, thereby serving as a primary cell culture that maintains features of the original host cells. Thus, it provided an ex vivo platform demonstrating occult HCV’s ability to infect the naïve PBMCs. Furthermore, the mitogen activation of PBMCs predisposed the cells to infection by wild-type HCV, simulating a true infection by the virus circulating in our patients. The obtained results also confirmed that other researchers have shown that circulating PBMCs are an extrahepatic site of virus replication in HCV-infected patients (25). In this study, naïve PBMC culture supported active HCV replication at low levels, although ex vivo activation of these cells was usually required to uncover the presence of the virus.

OCI is yet to be defined as either a new form of HCV infection or just a variation of chronic HCV. A study of OCI showed different variants of de novo HCV replicates in the stimulated T-cell culture in terms of buoyancy and nucleotide sequences (26). In the current study, a new variant of HCV, which was distinct from those viruses circulating in the host, has been detected in all the six different occult HCV sequences, as shown by the deletion of G nucleotide at a specific position of the 5’UTR. The mutation does not affect the overall molecular structures of the ribosomal entry sites for the initiation of viral translation and replication (Figure 3C). However, the same variant was also found among patients with CCI, suggesting that it is an adaptation of the occult virus to the PBMC environment permitting its persistence. Although the viral load is low, the HCV RNA can be recovered continuously from the PBMC culture of patients with either OCI or CCI. Therefore, it is evident that OCI possesses molecular mechanisms towards persistence similar to those of CCI.

## Conclusion

Taking together all evidence from the study, we concluded that seronegative OCI is an active form of infection. The virus is also infective and replicative ex vivo at a low level in PBMCs. Seronegative OCI may be a common occurrence among HD patients due to impaired immunity, older age and prolonged dialysis treatment, but the clinical significance of OCI warrants further studies to explore the exact extent of OCI among patients and to highlight the importance of incorporating HCV viral assays on PBMCs into the diagnostic algorithm in the era of direct-acting antiviral agents for the treatment of hepatitis C (27). Others have recommended methods of increasing the sensitivity of detecting the virus in OCI patients with ultracentrifugation of plasma rather than serum before performing PCR and developing more sensitive diagnostic tests (28). Nevertheless, awareness of OCI should be inculcated among health practitioners, to encourage both extra precautions for prevention and further clinical studies.

## Figures and Tables

**Figure 1:**
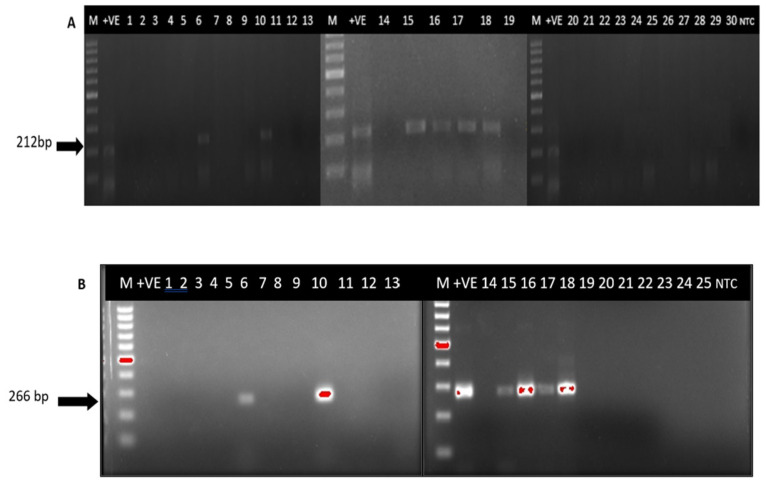
Detection of genomic and antigenomic HCV RNA in PBMC samples of haemodialysis patients who were also seronegative for anti-HCV antibodies. (A) The genomic HCV RNA was detected in six PBMC samples out of 30 samples. (B) Antigenomic HCV RNA was also detected in the six PMBC samples Notes: M = 100 bp DNA ladder (New England Biolabs, USA); +VE = positive control; NTC = no-template control; lane numbers indicate the sample’s number

**Table 1:** Primers used in the study for PCR amplification and DNA sequencing purposes

Target gene	Primer’s name	Purpose of the primer	Sequence (5’-3’)	Amplicon’s size (bp)	Reference
5’UTR^a^	HCV-F	Detection of genomic HCV RNA/First round	AGT GTT GTG CAG CCT CCA G	212	(15)
	HCV-R	Semi-nested PCR	ACT GCC TGA TAG GGT GCT TG		
	HCV-nF		CGG TGA GTA CAC CGG AAT TG	153	

	HCV-negF	Detection of anti-genomic HCV RNA	CAT GGT GCA CGG TCT ACG AGA CC	327	(14)
	HCV-negR		GGC GAC ACT CCA CCA TGA ATC AC		
	2NesF	Nested PCR	CTG TGA GGA ACT ACT GTC TT	266	
	2NesR		CTC GCA AGC ACC CTA TCA GG		

NS5B^b^	243-F	HCV subtyping	TGG GGA TCC CGT ATG ATA CCC GCT GCT TTG A	400	(16)
	242-R		GGC GGA ATT CCT GGT CAT AGC CTC CGT GAA		

Notes: a = 5’ untranslated region; b = nonstructural 5B

**Table 2:** Demographic and clinical characteristics of haemodialysis patients by the status of HCV infection

Variables	Non-infected (*n* = 24)	CCI (*n* = 10)	Seronegative OCI (*n* = 6)	*P*-value^b^
Age (years old)^a^	36.3 (9.6)	39.6 (8.4)	52.0 (10.7)	**0.019** c
Gender (female/male)	10/14	3/7	2/4	
Duration of dialysis (years)	3.5 (1.06)	5 (2.33)	4.3 (0.7)	**0.007** d
ALT (IU/L) (N = 0–55)	15.5 (6.3)	18.6 (4.7)	28.0 (19.6)	0.409^e^
AST (IU/L) (N = 5–34)	17.0 (6.0)	17.9 (4.3)	22.7 (11.4)	0.705^f^
Haemoglobin (g/dL) (N = 13.0–18.0)	10.5 (1.5)	10.5 (1.4)	10.8 (2.4)	0.902^g^
Serum creatinine (μmol/L) (N = 70–120)	534.7 (203.4)	657.1 (193.0)	647.8 (114.4)	0.192^h^

Notes:

a mean (SD);

b *P* < 0.05 is bolded;

c Kruskall-Wallis H(3) = 7.90;

d Kruskall-Wallis H(3) = 9.945; Pairwise comparisons with Bonferroni correction shows significant different between the non-infected group and CCI group (*P* = 0.024);

e Kruskall-Wallis H(3) = 1.788;

f Kruskall-Wallis H(3) = 0.700;

g One-way ANOVA, *F* score = 0.103;

h One-way ANOVA, F score = 1.726;

ALT = alanine transaminase; AST = aspartate transaminase; N = normal value

**Table 3:** Demographic, clinical and laboratory data of the patients with CCI and seronegative OCI

Sample name	Age (years old)	Sex	Duration of dialysis (years)	AST: ALT ratio	ALT a(IU/l)	Virus type (5’UTR)	Virus subtype (NS5B)	Viral load (log_10_ RNA copies/10^6^ cells)
*CCI* (*n* = 10)								
POS1B	33	M	6	0.9	16	3	UD	4.3
POS2B	41	F	5	1.0	22	3	UD	4.5
POS3B	34	M	4	0.9	14	3	UD	5.2
POS4B	43	F	10	1.1	15	1	1a	< 0.5
POS5B	47	F	3	0.9	17	3	UD	4.3
POS6B	29	M	4	0.9	18	6	UD	7.7
POS7B	31	F	5	1.0	20	3	3a	5.3
POS8B	38	M	3	0.9	16	3	3a	4.8
POS9B	41	M	2	1.1	17	3	3a	4.2
POS10B	59	M	7	0.9	31	3	3a	4.9

*OCI (n = 6)*								
HD6	45	M	4	1.1	16	3	UD	3.7
HD10	64	F	5	1.1	19	3	UD	3.0
bHD15	66	M	5	0.9	11	**3**	**1a**	4.3
HD16	56	F	3	0.7	**56**	3	UD	2.0
HD17	43	M	5	0.7	**55**	3	3a	3.7
HD18	38	M	4	1.1	11	3	3a	3.5

Notes: normal values of enzymes: ALT, 0–55; normal AST: ALT ratio, < 1;

a bold indicates a high ALT value;

b sample identified with mixed genotype infection;

M = male; F = female; AST = aspartate transaminase; ALT = alanine transaminase; UTR = untranslated region; NS5B = non-structural 5N; UD = undetermined

**Figure 1:**
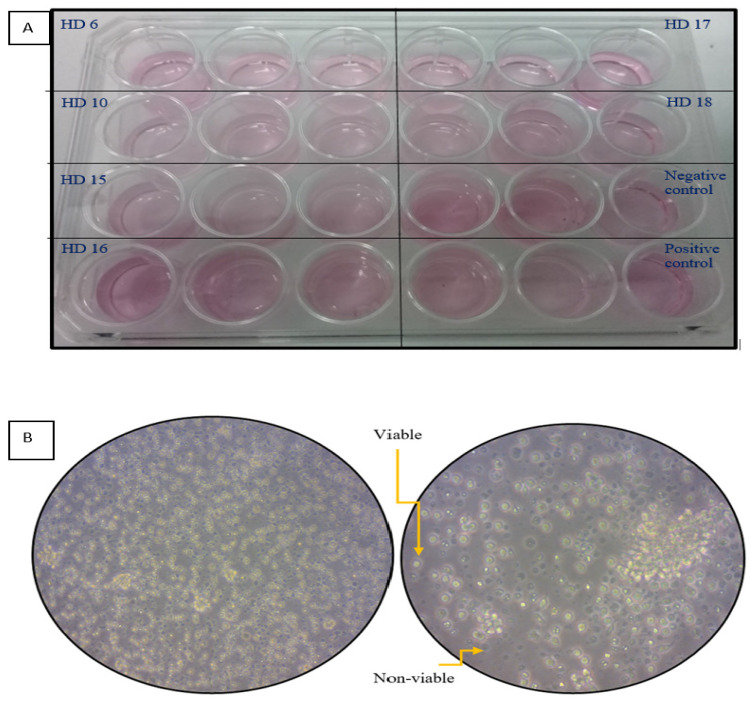
(A) Infectivity assay. Cells were aliquoted in a triplicate for each sample and incubated with the following inoculums: supernatants from the PBMCs of six occult HCV- infected patients, HCV-infected patients for positive control and healthy individual for negative control. (B) PHA-treated PBMC were observed using Digital Microscope (Dino Lite, Taiwan) in the magnification of 4× (left) and 10× (right)
